# Myelophthisic Anemia in a Patient with Lobular Breast Carcinoma Metastasized to the Bone Marrow

**DOI:** 10.7759/cureus.3541

**Published:** 2018-11-04

**Authors:** Muhammad H Khan, Abdurraoof Patel, Purav Patel, Poras Patel, Elizabeth Guevara

**Affiliations:** 1 Internal Medicine, The Brooklyn Hospital Center, Brooklyn, USA; 2 Hematology / Oncology, The Brooklyn Hospital Center, Brooklyn, USA

**Keywords:** ttp, thrombotic thrombocytopenic purpura, adamst13, a disintegrin and metalloproteinase with a thrombospondin type 1 motif (member 13), er, estrogen receptor, pr, progesterone receptor, her2, human epidermal growth factor receptor 2

## Abstract

Breast tumors have a predilection for metastasizing to the bone leading to cells being displaced by the cancer cells subsequently producing immature leukocytes and erythrocytes in the peripheral blood. We present a case of a 57-year-old female who was found to have myelophthisic anemia secondary to stage four lobular breast carcinoma metastasized to the bone marrow after being misdiagnosed as having thrombotic thrombocytopenia purpura. Diagnosis of myelophthisic anemia requires a thorough workup and treatment is based upon secondary management of the malignancy.

## Introduction

Approximately 252,710 new cases of invasive breast cancer were predicted to occur in the United States in 2017. From 2005 to 2014, the incidence of breast cancer among non-Hispanic blacks increased by 0.4% per year [1]. Breast tumors have a predilection for metastasizing to the bone. When metastasis to the bone marrow occurs, cells are displaced by the space-occupying cancer cells and this leads to immature leukocytes and erythrocytes in the peripheral blood [2-3]. Appearance of immature cells in the blood smear and evidence of bone marrow infiltration are sufficient for diagnosis of myelophthisic anemia [3-5].

## Case presentation

**Figure 1:**
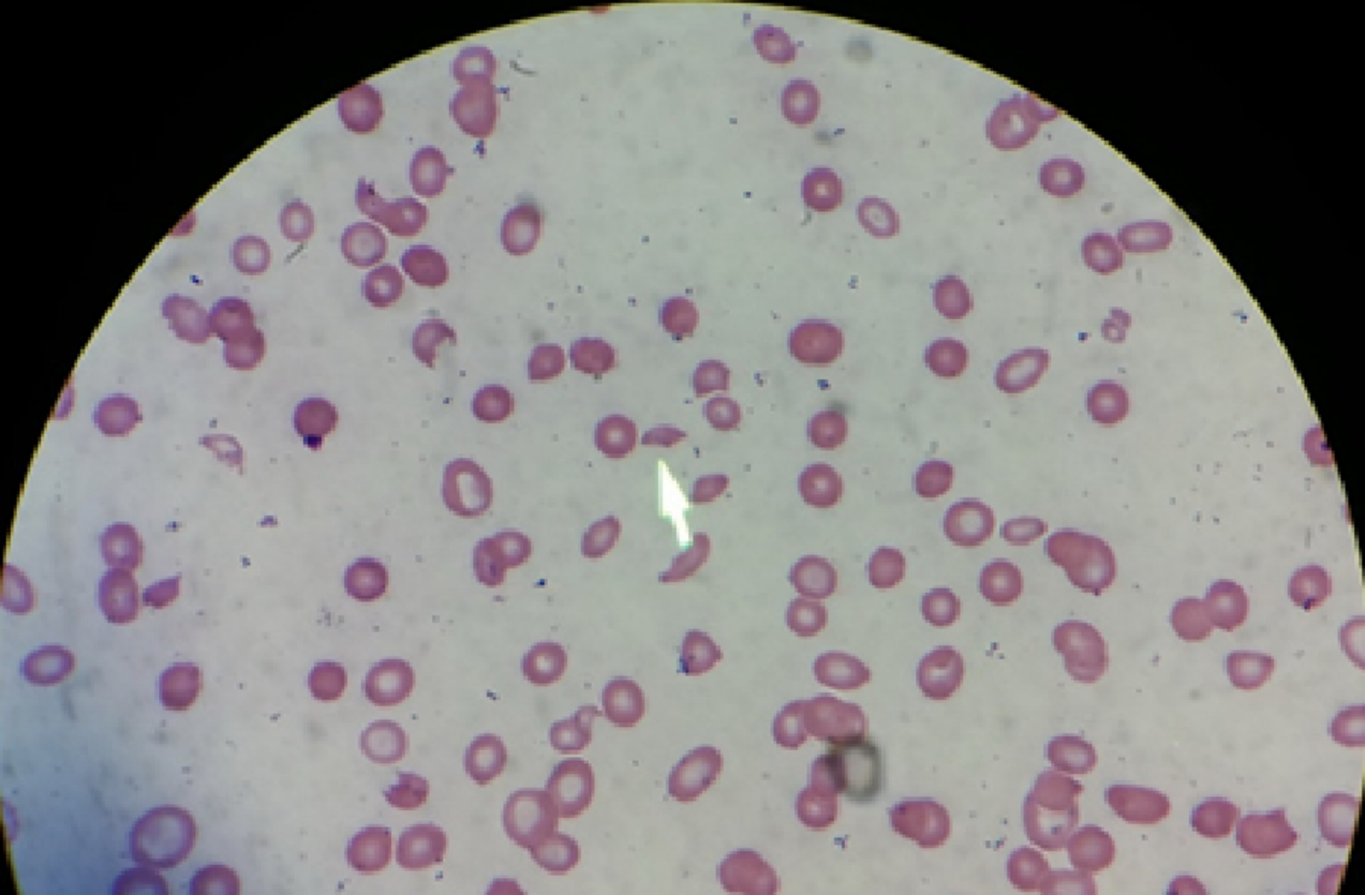
Fragmented red blood cells (schistocytes), as shown by the arrow.

A 57-year-old African American female with a history of diabetes presented to the hospital with severe anemia and acute change in mental status. On physical examination, the patient was noted to be lethargic and had right-sided facial drooping, right-sided tongue deviation, right-sided gaze preference, with right-sided body strength significantly diminished compared to the left. Initial laboratory results, reported in Table 1, showed severe anemia and thrombocytopenia (Hb 2.3 g/dL, Hct 8 %, Plt 15,000/cmm), and mild acute kidney injury (CrCl 101 mL/min). Numerous fragmented red blood cells (RBCs) (schistocytes) were noted in the peripheral blood smear (Figure 1). Repeated peripheral blood smears persistently showed poikilocytosis, nucleated RBCs, immature myeloid cells, and teardrop cells. Thrombotic thrombocytopenic purpura (TTP) was suspected due to classic presentation: microangiopathic hemolytic anemia, thrombocytopenia, acute kidney injury, altered mental status, and a low grade fever.

**Table 1:** Labs obtained upon day of admission. Na, sodium; K, potassium; Cl, chloride; CO_2_, bicarbonate; BUN, blood urea nitrogen; Cr, creatinine; Total bili, total bilirubin; Direct bili, direct bilirubin; AST, aspartate aminotransferase; ALT, alanine aminotransferase; Alb, albumin; ALP, alkaline phosphatase; TPro, total protein; WBC, white blood cell; Hb, hemoglobin; Hct, hematocrit; Plt, platelet; MCV, mean corpuscular volume; MCH, mean corpuscular hemoglobin; MCHC, mean corpuscular hemoglobin concentration; RDW, red blood cell distribution width; Auto neutro, auto neutrophil; Auto lymph, auto lymphocytes; Auto mono, auto monocytes; Auto baso, auto basophil; Auto eos, auto eosinophil; Retic count, reticulocyte count; LDH, lactate dehydrogenase.

Laboratory	Value
Na	133 mmol/L
K	4.7 mmol/L
Cl	102 mmol/L
CO_2_	13 mmol/L
BUN	33 mg/dL
Cr	0.7 mg/dL
Total Bili	1.3 mg/dL
Direct Bili	0.8 mg/dL
AST	152 U/L
ALT	44 U/L
Alb	3.2 g/dL
Alk Phos	644 U/L
Total Pro	7.3 g/dL
Lactate	8.1 mmol/L
Haptoglobin	8.0 mg/dL
WBC	9.3 K/cmm
Hb	2.3 g/dL
Hct	8.0%
Plt	15 K/cmm
MCV	80 fL
MCH	25 pg
MCHC	31.6 g/dL
RDW	29.3%
Auto neutro	54.8%
Auto lymph	35.3%
Auto mono	9.2%
Auto baso	0.5%
Auto eos	0.2%
Retic count	6.1%
LDH	1092 U/L

**Figure 2:**
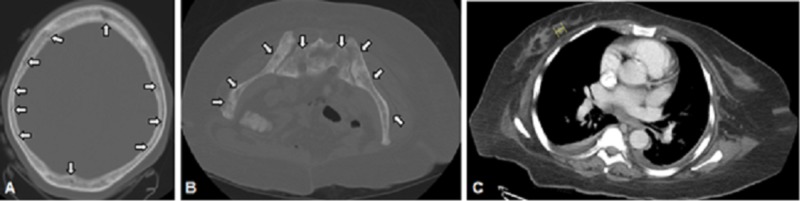
A) White arrows point to mixed lytic and sclerotic lesions of the calvarium found on computed tomography (CT) scan of the head without contrast. B) White arrows point to osteoblastic lesions of the pelvis found on CT scan of the abdominopelvic region without contrast. C) Yellow marker shows 1.2 cm ovoid soft tissue nodular opacity at the 6 o’clock position of the right breast.

**Figure 3:**
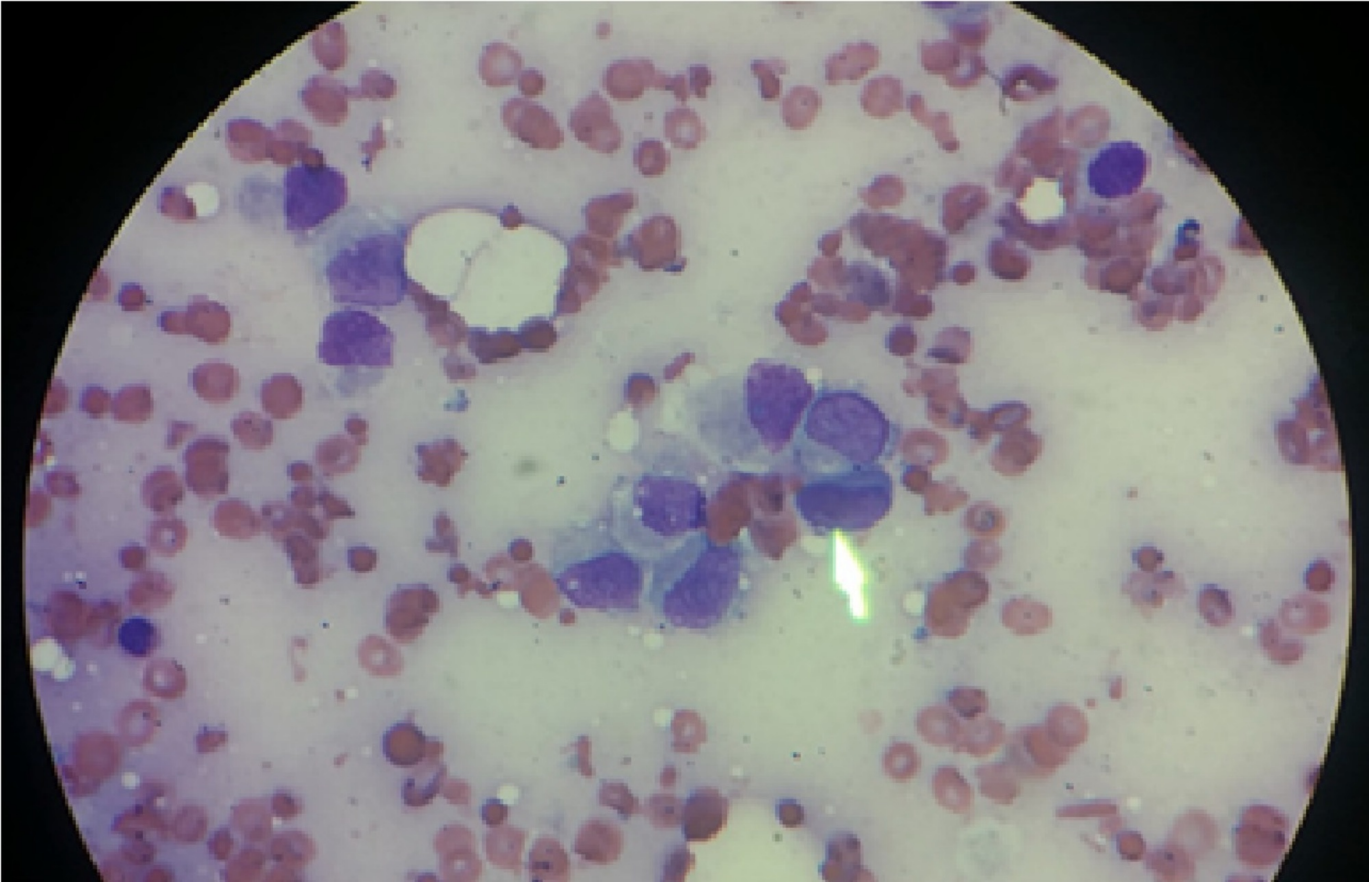
Atypical tumor cells, as shown by the arrow.

The patient was started on daily plasmapheresis and steroids for a presumed diagnosis of TTP. However, after ADAMTS13 result came back negative, plasmapheresis was stopped and the steroid was tapered. On further evaluation, computed tomography (CT) scan of the head revealed mixed sclerotic and lytic lesions in the calvarium (Figure 2A), diffuse osteoblastic pelvic lesions (Figure 2B), and a 1.2-cm ovoid soft tissue nodular opacity in the 6 o’clock position of the right breast (Figure 2C). Subsequent tests including bone marrow aspiration yielded a dry tap further solidifying the concern for bone marrow infiltrative disease. Bone marrow biopsy from the ischial bone showed many atypical cells (Figure 3), which were highly suggestive of carcinoma and the immunohistochemistry report was consistent with metastatic lobular breast carcinoma with the tumor cells staining positive for both estrogen receptor (ER) and progesterone receptor (PR), and negative for HER2. She was started on combination therapy with letrozole (aromatase inhibitor) and palbociclib (cyclin-dependent kinase inhibitor). The patient had significant clinical and hematological improvement within few days after starting the combination therapy. Her repeat laboratory test results are reported in Table 2. Two months later, the patient presented to the emergency room with deteriorated clinical status and severe pancytopenia. Despite aggressive measurements, she succumbed to her illness.

**Table 2:** Labs after combination therapy. Na, sodium; K, potassium; Cl, chloride; CO_2_, bicarbonate; BUN, blood urea nitrogen; Cr, creatinine; Total bili, total bilirubin; Direct bili, direct bilirubin; AST, aspartate aminotransferase; ALT, alanine aminotransferase; Alb, albumin; ALP, alkaline phosphatase; TPro, total protein; WBC, white blood cell; Hb, hemoglobin; Hct, hematocrit; Plt, platelet; MCV, mean corpuscular volume; MCH, mean corpuscular hemoglobin; MCHC, mean corpuscular hemoglobin concentration; RDW, red blood cell distribution width; Auto neutro, auto neutrophil; Auto lymph, auto lymphocytes; Auto mono, auto monocytes; Auto baso, auto basophil; Auto eos, auto eosinophil; Retic count, reticulocyte count; LDH, lactate dehydrogenase.

Laboratory	Value
Na	145 mmol/L
K	3.6 mmol/L
Cl	111 mmol/L
CO_2_	25 mmol/L
BUN	15 mg/dL
Cr	0.8 mg/dL
Total Bili	1.0 mg/dL
Direct Bili	0.8 mg/dL
AST	87 U/L
ALT	16 U/L
Alb	2.7 g/dL
Alk Phos	181 U/L
Total Pro	7.7 g/dL
Lactate	1.6 mmol/L
Haptoglobin	12.0 mg/dL
WBC	11.4 K/cmm
Hb	7.7 g/dL
Hct	25.0%
Plt	140 K/cmm
MCV	91 fL
MCH	27 pg
MCHC	29.9 g/dL
RDW	21.7%
Auto neutro	58.6%
Auto lymph	24.5%
Auto mono	16.4%
Auto baso	0.5%
Auto eos	0.0%
Retic count	3.8%
LDH	764 U/L

## Discussion

Myelophthisic anemia, also known as myelophthisis, is a type of bone marrow failure that results from displacement of bone marrow precursor cells from a multitude of etiologies such as storage diseases, fungal infections, or metastatic neoplasms. Myelophthisic anemia is a rare occurrence as it is found in less than 10% of patients with metastatic malignancies such as lung cancer, breast cancer, prostate cancer, or sarcomas [4]. These malignancies spread and invade the bone marrow with space-occupying lesions, replacing the hematopoietic stem cells in the marrow and ultimately resulting in pancytopenia and extramedullary hematopoiesis. These lesions can be identified by a peripheral blood smear demonstrating a leukoerythroblastic picture with immature and abnormal RBCs in the form of schistocytes with myeloid precursors [2, 4].

Due to discovery in the advanced stages of the malignancy, myelophthisic anemia is thought to be resistant to treatment and considered as a poor prognostic sign [3-4]. Previously held beliefs that some cases of metastatic breast cancer had undergone remission with manipulation of hormones led to androgen administration, adrenalectomy, hypophysectomy, and even castration. Unfortunately, these radical procedures were only found to provide temporary relief [3]. With the advancement of medical therapy, current treatment for myelophthisic anemia is by directly treating the primary cancer. Treating this patient’s ER-positive Stage IV lobular breast carcinoma with letrozole and palbociclib was found to initially improve the patient’s symptoms but there was no mortality benefit and the patient passed away after two months of chemotherapy. No prior studies have suggested chemotherapy regimen amelioration in both morbidity and mortality and the recommended duration and prognosis of the treatment is yet to be determined [5].

This patient’s distinct presentation was highly indicative of TTP on initial evaluation. Unable to obtain history from the patient and relying solely on papers received from the patient’s nursing home resulted in reflexive decision-making leading to the patient undergo superfluous sessions of plasmapheresis. A more careful approach is required to rule out all other possible etiologies of this patient’s symptoms and laboratory findings and, thereafter, consider TTP as a diagnosis of exclusion.

## Conclusions

Myelophthisic anemia is not an obvious diagnosis and it is affirmed through laboratory findings, peripheral blood smear, and bone marrow biopsy. Treatment and prognosis of myelophthisic anemia is variable as it is based upon secondary management of the malignancy.
